# Inhibition of the Axl pathway impairs breast and prostate cancer metastasis to the bones and bone remodeling

**DOI:** 10.1007/s10585-021-10093-z

**Published:** 2021-03-31

**Authors:** Mai Tanaka, Samantha S. Dykes, Dietmar W. Siemann

**Affiliations:** 1 Department of Radiation Oncology, College of Medicine, University of Florida, Gainesville, FL 32610 USA; 2 GenCure, a Subsidiary of BioBridge Global, San Antonio, TX 78201 USA

**Keywords:** Axl, Bone metastasis, Receptor tyrosine kinase, Osteoclastogenesis

## Abstract

Approximately 90% of cancer-related deaths result from cancer metastasis. In prostate and breast cancers, bone is the most common site of cancer cell dissemination. Key steps in the metastatic cascade are promoted through upregulation of critical cell signaling pathways in neoplastic cells. The present study assessed the role of the receptor tyrosine kinase Axl in prostate and breast cancer cell metastasis to bones using (i) Axl knockdown neoplastic cells and osteoclast progenitor cells *in vitro*, (ii) intracardiac injection of Axl knockdown tumor cells *in vivo,* and (iii) selective Axl inhibitor BGB324. Axl inhibition in neoplastic cells significantly decreased their metastatic potential, and suppression of Axl signaling in osteoclast precursor cells also reduced the formation of mature osteoclasts. *In vivo*, Axl knockdown in prostate and breast cancer cells significantly suppressed the formation and progression of bone metastases. Hence, therapeutic targeting of Axl may impair tumor metastasis to the bones through neoplastic and host cell signaling axes.

**Supplementary Information:**

The online version contains supplementary material available at 10.1007/s10585-021-10093-z.

## Background

Cancer is the second leading cause of death in the United States. Prostate and breast cancer are the most commonly diagnosed cancers and the second leading cause of cancer deaths for both men and women in the United States, respectively [1]. Tumor metastasis, the spread of cancer cells to secondary sites, is the primary cause of cancer-related mortality [2]. The process of tumor cell dissemination involves several critical molecular and cellular steps. Each step of the metastatic cascade is essential: migration and invasion are necessary at both the initiation and the end of the metastatic cascade [3]; survival is critical during the circulation of tumor cell in the blood stream [4]; the metastatic colonization reflects the combined post-extravasation events [5]; and the initiation of angiogenesis is required for the successful outgrowth of distant neoplastic lesions [6–8].

In 1889, Stephen Paget proposed the “seed and soil” hypothesis, which suggests that the pattern of metastasis is not due to chance, and that tumor cells (the seeds) preferentially grew in the fertile microenvironment of certain organs (the soil) [9]. Indeed, bone is the most common site of metastasis for both prostate and breast cancers [10]. Successful development of metastatic lesions in the bone involves multiple steps, including (1) colonization of the bone, (2) dormancy and adaptation into the new microenvironment, (3) reactivation and proliferation from the dormant state, and (4) cancer-induced bone remodeling [11].

When tumor cells colonize the bone and have reactivated from the dormant state, they can initiate abnormal bone remodeling, also known as the vicious cycle. While physiologic bone remodeling cycle is tightly regulated, tumor cells disrupt the homeostasis between the osteoblasts and osteoclasts that are involved in bone formation and resorption, respectively [12]. For example, breast cancer bone metastases are often characterized by its osteolytic lesions [13], where breast cancer cells secrete factors that directly and indirectly stimulate the osteoclast functions [6]. Consequently, breast cancer cells can promote osteoclastogenesis, a process in which mature osteoclasts resorb the bone, releasing matrix-stored growth factors to stimulate tumor growth and further osteolysis. This process sustains itself through its positive-feedback mechanism and increases tumor burden. Hence, stromal cells in the metastatic tumor microenvironment also contribute to tumor progression.

Receptor tyrosine kinases (RTKs) frequently are overexpressed in cancer cells and dysregulate a number of signaling pathways that are involved in the metastatic cascade [14, 15]. One signaling pathway of considerable interest is the receptor tyrosine kinase Axl. Axl belongs to the Tyro-3, Axl and Mer (TAM) subfamily of the RTK [16–18]. Axl is overexpressed in many cancer types and has been associated with tumor progression, cancer stem cell phenotype, therapeutic resistance, immune suppression, and poor clinical prognosis and outcome [19–26]. Previous preclinical studies have shown that genetic suppression of Axl by short hairpin RNA (shRNA) knockdown decreased cell migration and invasion in colorectal and cervical cancer cell lines [27]. In a pancreatic cancer cell line, Axl knockdown by shRNA decreased GTP-bound forms of Rho and Rac, which control a number of cytoskeletal dynamics including cell migration [28, 29].

In addition to neoplastic cells, Axl is expressed on stromal cells [30–32]. For example, pharmacologic inhibition of Axl on endothelial cells decreases angiogenic functions [30]. Axl inhibition also decreases tumor cell-induced angiogenesis and recruitment of CD31-expressing cells to the tumor mass [33–36]. Therefore, we hypothesized that Axl-expressing tumor cells and stromal cells could promote a number of different steps in the metastatic cascade. Overall, the goal of the present study was to assess the role of Axl in neoplastic and stromal cells on prostate and breast cancer bone metastasis as bone is the most common site of metastasis for these tumor types.

## Methods

### Cell lines and cell culture

MDA-MB-231 and Raw264.7 cells were cultured in Dulbecco’s Modified Eagle Medium (DMEM). PC3ML is a metastatic subline of human prostate cancer cells (PC3), isolated through *in vivo* selection of bone metastases [37]. PC3ML cells were cultured in Ham’s F12 media. DU-145 cells were cultured in Eagle’s Minimum Essential Medium. Media were supplemented with 10% fetal bovine serum, 1% L-glutamine, and 1% penicillin–streptomycin. Cells were maintained at 37 °C in a humidified atmosphere of 5% CO_2_. Mycoplasma tests were performed using MycoAlert Mycoplasma Detection Kit (Lonza).

### Reagents

Mouse recombinant RANK-L (Catalog #: 462-TEC) and MCP-1 (Catalog #: 479-JE) were obtained from R&D Systems (Minneapolis, MN). BGB324 was obtained from Selleckchem (Houston, TX). BGB324 was aliquoted in sterile DMSO and stored at −20 °C.

### Generation of stable Axl knockdown cell lines using shRNA

Axl knockdown MDA-MB-231, PC3ML and Raw264.7 cell lines were generated with Mission Lentivirus Transduction particles (Sigma-Aldrich). Lentiviral particles containing scrambled non-silencing shRNA (shSCM, SHC202V) or Axl shRNA (MDA-MB-231 shAXL#1: TRCN0000001039, shAXL#2: TRCN0000001040; PC3ML shAXL#1: TRCN0000001039, shAXL#2: TRCN0000001040; DU-145 shAXL#1: TRCN0000001040, shAXL#2: TRCN0000001041; and Raw264.7: shAXL#1: TRCN0000322131, shAXL#2: TRCN0000322132) were transduced in respective cells. When cells reached 50–60% confluence, cells were infected with 6 μg/mL polybrene (Millipore) and lentiviral particles. After 48 h, the cells were selected with 3.6 μg/mL of puromycin (Thermo Fisher Scientific). Knockdown cells were maintained under puromycin selection for the duration of the experiments.

### Western blot analysis

Cells were lysed on ice by scraping into RIPA buffer (50 mM Tris–HCl, pH8.0; 150 mM NaCl; 0.1% SDS; 1% NP-40; 0.25% Sodium deoxycholate, and 1 mM EDTA) containing protease inhibitor (Sigma Aldrich), 1 mM NaF, and 1 mM Na_3_VO_4_. Protein concentration was measured by BCA assay, and equal amounts of protein were diluted and boiled in laemmli loading buffer. Whole cell lysates were separated by electrophoresis on SDS-PAGE gels and then transferred to a PVDF membrane. Membrane was blocked for 1 h with 5% BSA in TBS-T (20 mM Tris; 137 mM NaCl; 0.1% Tween-20, pH 7.5), and with a primary antibody diluted in 5% BSA in TBS-T overnight. Membrane was washed for 10 min with TBS-T for three times and incubated with secondary antibody diluted in TBS (20 mM Tris, 137 mM NaCl, pH 7.5) for at least 1 h. The signal was detected with an enhanced chemiluminescence substrate (Catalog #: RPN2209, GE Healthcare) and imaged on the Amersham Imager 680 (GE Healthcare). Primary antibodies: *human Axl* (Dilution: 1:1 000, Catalog #: 8661S, Cell Signaling Technologies) and β-*actin* (Dilution 1:20 000, Catalog #, A1978, Sigma-Aldrich). Horseradish peroxidase-conjugated secondary antibodies: Goat anti-mouse IgG (Dilution: 1:10 000, Catalog #:115-035-003, Jackson ImmunoResearch), mouse anti-human IgG (Dilution: 1:20 000, Catalog #:209-035-088, Jackson ImmunoResearch), and rabbit anti-human IgG (Dilution: 1:10 000, Catalog #: 309-035-003, Jackson ImmunoResearch).

### RNA isolation and qPCR analysis

Total RNAs were isolated from cells using the Direct-zol RNA kit (Zymo-Research) according to the manufacturer’s instructions. RNAs were reverse transcribed into cDNAs by using the TaqMan Reverse Transcription reagents (Invitrogen). Real-time PCR was performed on the StepOne Real-Time PCR Systems (Applied Biosystems) using SYBR Green PCR Master Mix (Applied Biosystems). The gene-specific primer sets were used at a final concentration of 300 nM. All qPCR assays were performed in triplicates in three independent experiments. Relative expression levels of target genes were normalized to the mean cycle threshold (Ct) values of target gene to the mean Ct values of the housekeeping *GAPDH* gene. The relative expression levels were determined as 2^−ΔCt^. The primers (Table 1) used in this study were synthesized by IDT.

**Table 1:** Primer sequences for qPCR

Gene	Forward sequence	Reverse sequence
Mouse AXL	5′-GGAAAGAGGTGAACTGGTAGTC-3’	5′- CCATGACGTCTCGTAGTTTCTC-3’
Mouse MCP-1	5′-CTTCTGGGCCTGCTGTTCA-3’	5′-CAGCCTACTCATTGGGATCA-3’
Mouse GAPDH	5′-TATGTCGTGGAGTCTACTGGT-3’	5′-GAGTTGTCATATTTCTCGT-3’

### Transwell chamber assays

MDA-MB-231, PC3ML, DU-145, and Raw264.7 cell migrations were examined using a transwell insert with 8 μm pores membrane (BD, Franklin Lakes, NJ). Cells (MDA-MB-231: 5 × 10^3^ cells; PC3ML: 5 × 10^3^ cells; DU-145: 5 × 10^3^ cells; and Raw264.7: 5 × 10^3^ cells) were seeded in the inserts with complete medium. After 24 h, the cells on the underside of the insert were stained with crystal violet and counted. In the invasion assay, inserts were coated with 10% Matrigel in serum-free medium for 1 h. Cells (MDA-MB-231: 1 × 10^4^ cells; PC3ML: 1 × 10^4^ cells; DU-145: 2 × 10^4^ cells; and Raw264.7: 2 × 10^4^ cells) were suspended in serum-free media and loaded into the insert. Complete medium was used in the lower chamber as a chemo-attractant. After 24 h, cells were fixed and stained with 100% EtOH and crystal violet.

For transwell migration and invasion assays using the selective Axl inhibitor, BGB324, MDA-MB-231 and PC3ML cells were resuspended in complete or serum-free media containing vehicle control or BGB324 for migration (MDA-MB-231: 5 × 10^3^ cells; and PC3ML: 5 × 10^3^ cells) and invasion (MDA-MB-231: 1 × 10^4^ cells; and PC3ML: 1 × 10^4^ cells) assays, respectively. Complete medium containing vehicle control or BGB324 was used in the lower chamber. All migration/invasion assays were performed in triplicates in three independent experiments. After 24 h, cells were fixed and stained with 100% EtOH and crystal violet.

### Cell proliferation assay (Trypan Blue)

Tumor cell proliferation was assayed using trypan blue dye. PC3ML and DU-145 cells (shSCM or shAXL) were seeded at a concentration of 1 × 10^4^ cells/60 mm^2^ dish (Day 0). Total number of viable cells were measured using the trypan blue dye on Day 1, 2, 3, 5, and 7 (PC3ML) and Days 1, 2, 3, 4, and 7 (DU-145). All cell proliferation assays were performed in triplicates in three independent experiments.

### Cell proliferation assay (CCK-8)

Tumor cell proliferation was assayed using CCK-8 (Dojindo Molecular Technologies, Rockville, MD). MDA-MB-231 and DU-145 cells (shSCM or shAXL) were seeded at a concentration of 1.5 × 10^3^ cells/well in 96-well flat-bottom plate (Day 0). Cell proliferation was assessed according to the manufacturer’s instructions at 0, 24, 48, and 72 h. The effect of BGB324 on cell proliferation was also assessed on MDA-MB-231 and PC3ML cells. Cells were seeded at a concentration of 1.5 × 10^3^ cells/well in 96-well flat-bottom plate. After 24 h, culture medium was replaced with media containing vehicle control, 0.1 μM, 0.5 μM, 1.0 μM, 2.0 μM, or 5.0 μM BGB324. Cell proliferation was assessed by CCK-8 according to the manufacturer’s instructions at 24 h, 48 h, and 72 h after BGB324 treatment. CCK-8 assays were performed in triplicates in three independent experiments.

### Osteoclast formation assay

For osteoclast formation assay using Raw264.7 cells, Raw264.7 cells (shSCM or shAXL) were seeded at a concentration of 2 × 10^4^ cells/well in 24-well plate (Day 1). Cells were treated with 35 ng/mL RANK-L in complete DMEM, and the media were replaced every two days until day 6. For the treatment with BGB324, Raw264.7 cells were seeded at the concentration of 2 × 10^4^ cells/well in 24-well plate (Day 1). Cells were treated with 35 ng/mL RANK-L in complete DMEM. After 24 h, vehicle control or BGB324 were added to the culture media. The media containing RANK-L and/or BGB324 were replaced every two days until day 6.

For the osteoclast formation assay using primary osteoclast precursor cells, bone marrow derived cells (BMDCs) were isolated from 6 to 8 week-old female BALB/c mice by flushing the bone marrow of tibiae and femurs with a 26-G needle. The BMDCs were spun down at 300 × *g* for 5 min to remove cell debris. Cells were resuspended and seeded in alpha-Minimum Essential Medium with 10% FBS and 50 ng/mL MCP-1 for three days. On day 3, supernatant was removed and adherent, osteoclast precursor cells, were used for the osteoclast formation assay. Primary osteoclast precursor cells were seeded at a concentration of 2.5 × 10^5^ cells/well in media containing 35 ng/mL RANK-L and 50 ng/ml MCP-1. After 24 h, vehicle control or BGB324 was added to the culture media. The media were replaced every two days with RANK-L and MCP-1, with or without BGB324, until day 6.

At endpoint, cells were fixed and stained for Tartrate resistant acid phosphatase (TRAP) using the Acid Phosphatase, Leukocyte Kit (Sigma-Aldrich) according to the manufacturer’s instructions. Multinucleate (> 3 nuclei) and TRAP-positive osteoclasts were quantified and imaged using Leica DM4000 B LED microscope. All osteoclast formation assays were performed in triplicates in three independent experiments.

#### Resorption pit assay

Bovine cortical bone slices (Immunodiagnostic Systems) were sterilized with 70% ethanol for 5 min, followed by 10% penicillin–streptomycin for 5 min. The bones were rinsed twice with complete DMEM and incubated overnight at 37 °C. In a 24 well plate, Raw264.7 cells were seeded on the bone slices at a concentration of 2 × 10^4^ cells/well with 35 ng/mL RANK-L. The medium was replaced every two days. On day 6, cells on the bone slices were removed using a cotton swab. Bone slices were stained using toluidine blue solution (1% toluidine, 0.5% tetraborate in water) for 5 min and rinsed in PBS three times. The resorption pits were quantified and imaged using Leica DM4000 B LED microscope. Resorption pit assay was performed in three independent experiments.

#### Cytokine microarray

Osteoclast precursor cells (shSCM or shAXL) were seeded in a 60-mm dish. When the cells reached 50–60% confluence, the medium was replaced with serum free medium. Cell conditioned media were harvested 24 h later, centrifuged at 300 × *g* for 5 min to remove cell debris. Protein concentration in the conditioned media from each sample were measured by BCA for normalization of secreted levels. Cytokines secreted in the conditioned media were measured using the murine XL cytokine array (R&D Systems, Minneapolis, MN), according to the manufacturer’s instructions. Briefly, the blot was incubated with conditioned media. After washing and incubating the blot with HRP-conjugated secondary antibody, ChemiReagent Mix was used for chemiluminescent detection on the Amersham Imager 680 (GE Healthcare).

#### Intracardiac assay

Luciferase-labeled shSCM or shAXL MDA-MB-231 cells (2 × 10^6^ cells/mL) and PC3ML cells (10^6^ cells/mL) were suspended in a sterile saline, containing 150 μg/mL *D*-luciferin. Female and male athymic mice were randomized into three groups for injection of MDA-MB-231 and PC3ML cells, respectively (MDA-MB-231: shSCM, *n* = 8; shAXL#1, *n* = 8; shAXL#2, *n* = 10; PC3ML: shSCM, *n* = 10; shAXL#1, *n* = 9; shAXL#2, *n* = 10). To perform the injection using a closed-chest technique, a cell suspension (0.1 mL) was inoculated into the left ventricle of the heart of anesthetized athymic female nude mice using a 28-gauge needle. Anesthesia was induced with 1–3% isoflurane in oxygen in an induction chamber equipped with a scavenger system. Verification of a successful intracardiac inoculation was confirmed by bioluminescent imaging (BLI) using the Xenogen IVIS imaging system, immediately after injection and observing that luciferase-labeled cells have dispersed throughout the body rather than remaining in the thoracic area. Bone metastasis was monitored on a weekly basis using the Xenogen IVIS imaging system by measuring the photon flux 15 min after intraperitoneal injection of *D*-luciferin.

#### Statistical analysis

Data are expressed as means ± SEM. One-way ANOVA was applied unless otherwise noted. All statistical analysis was performed using GraphPad Prism 5.0 software (San Diego, CA). A threshold of *P* < 0.05 was designed as statistically significant.

## Results

### Axl is important mediator of tumor cell migration and invasion

**Fig. 1:**
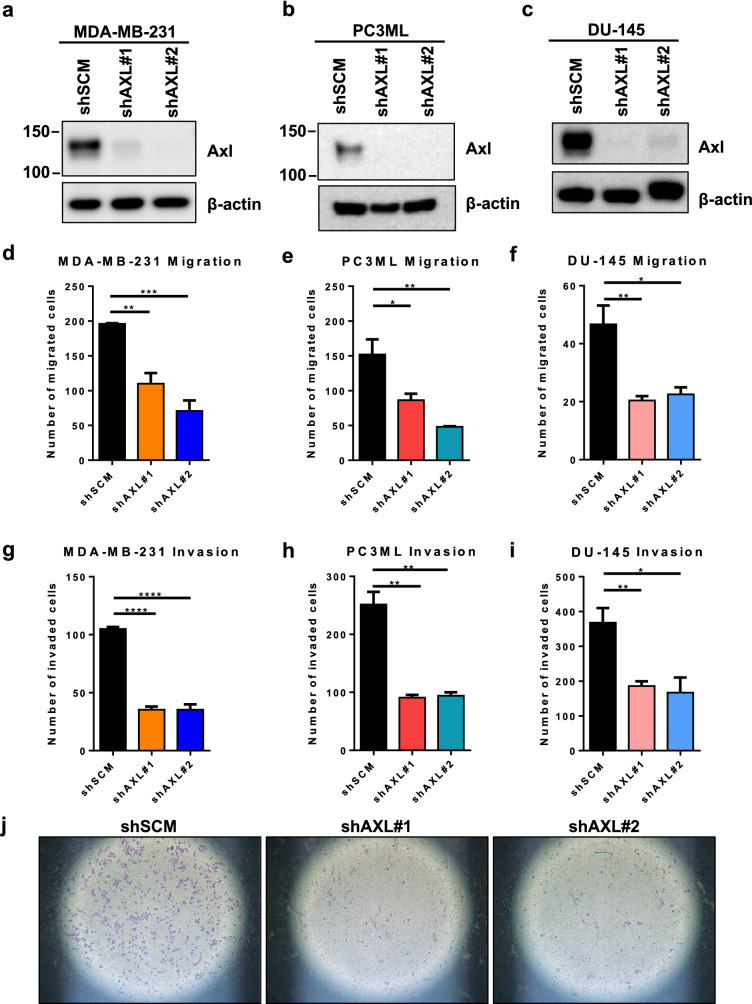
Axl knockdown in breast and prostate cancer cells decreases migratory and invasive capacities. Axl was genetically inactivated by shRNA in MDA-MB-231 (**a**), PC3ML (**b**), and DU-145 (**c**) cell lines and two knockdown clones (shAXL#1 and shAXL#2) were selected by Western blot for further studies. Tumor cells were seeded in the transwell migration or invasion chambers. The number of migrated (**d**–**e**) or invaded (**g**–**i**) tumor cells were counted 24 h later. **j** Representative images of invasion chambers that were seeded with shSCM or shAXL DU-145 cells. Results are the mean and standard error values of three independent experiments. *p < 0.05, **p < 0.01, ***p < 0.0001, ****p < 0.00001

**Fig. 2:**
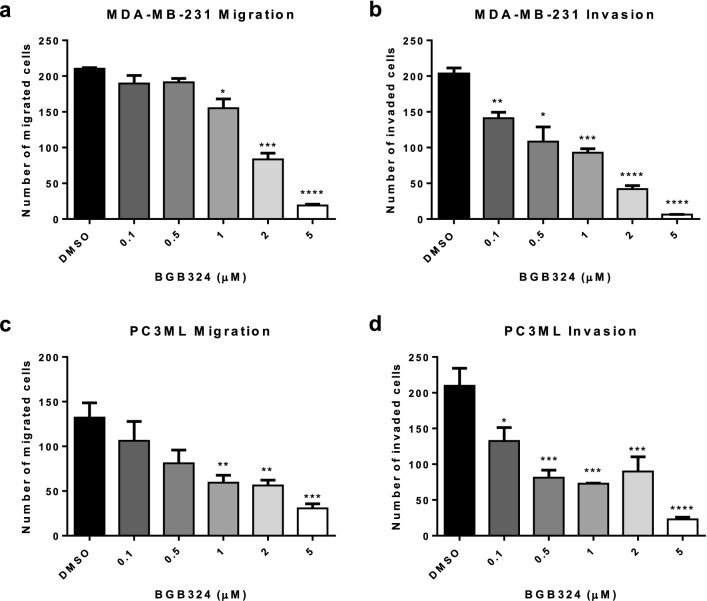
A selective Axl inhibitor, BGB324, impairs tumor cell migration and invasion. MDA-MB-231 and PC3ML cells were seeded in the transwell migration or invasion chambers with varying concentrations of BGB324. The number of migrated (**a**, **c**) or invaded (**b**, **d**) tumor cells were counted 24 h later. Results are the mean and standard error values of three independent experiments. *p < 0.05, **p < 0.01, ***p < 0.0001, ****p < 0.00001

To determine the role of Axl in tumor cell metastatic phenotypes, human breast (MDA-MB-231) and prostate cancer cell lines (PC3ML and DU-145) were transduced with lentiviral shRNA against scrambled non-target sequence (shSCM) or AXL (shAXL), and the transduction efficiency was confirmed by Western Blot (Fig. 1a–c). When assessed in transwell chamber assays, Axl knockdown tumor cells showed significantly decreased tumor cell migration compared to their parental counterparts (Fig. 1d–f). Similarly, Axl knockdown significantly impaired breast tumor cell invasion in both knockdown clones (Fig. 1g–j). To determine whether Axl knockdown impairs tumor cell proliferation, direct counting by trypan blue and CCK-8 assay were used. In both assays, there was no difference in cell proliferation between shSCM and shAXL cells (Supplementary Fig. 1). To assess the efficacy of pharmacologic inhibition of the Axl pathway on metastatic phenotype, a selective small molecule inhibitor against Axl, BGB324, was used. BGB324 significantly decreased MDA-MB-231 and PC3ML cell migration and invasion in a dose-dependent manner (Fig. 2). BGB324 had no significant impact on cell proliferation at 24 h; however, cell proliferation halted at 5 μM over 48 h and 72 h time course (Supplemental Fig. 2).

### Axl inhibition decreases breast cancer bone metastases

**Fig. 3:**
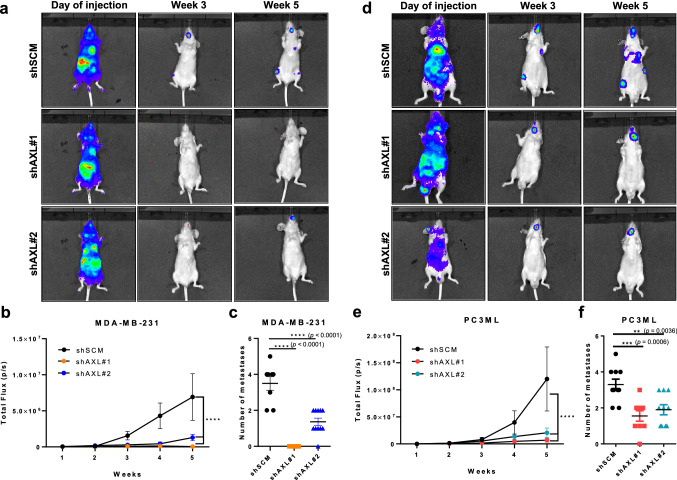
Axl knockdown in MDA-MB-231 and PC3ML cells decreases metastasis to the bone. shSCM or shAXL MDA-MB-231 or PC3ML cells, expressing luciferase, were inoculated intracardiacally into 6–8 week-old female and male athymic nu/nu mice, respectively. Tumor seeding and progression was monitored via weekly bioluminescence imaging (BLI). Five weeks later, mice were euthanized. Representative bioluminescence images of mice inoculated with shSCM or shAXL MDA-MB-231 (**a**) and PC3ML (**d**) cells at the time of injection, week 3, and week 5. Total flux of intracardiacally injected shSCM and shAXL MDA-MB-231 (**b**) and PC3ML (**e**) cells were tracked over 5 weeks. Number of macroscopic metastases in mice inoculated with either shSCM or shAXL MDA-MB-231 (**c**) and PC3ML (**f**) cells at endpoint. MDA-MB-231: shSCM, *n* = 8; shAXL#1, *n* = 8; shAXL#2, *n* = 10; PC3ML: shSCM, *n* = 10; shAXL#1, *n* = 9; shAXL#2, *n* = 10. **p = 0.0036, ***p = 0.0006, ****p < 0.00001

Because bone is the most common site of metastasis for both breast and prostate cancers, we also evaluated the effect of Axl knockdown on bone metastasis. The shSCM or shAXL tumor cells stably expressing luciferase were injected intracardiacally into nude mice and monitored weekly by bioluminescence imaging. After 5 weeks, both of the shAXL MDA-MB-231 cell lines showed a significantly decreased total fluorescence flux compared to the shSCM MDA-MB-231 cells (Fig. 3a and b) and resulted in significantly fewer bone metastatic lesions (Fig. 3c). Similarly, intracardiac inoculation of shAXL PC3ML cells produced significantly fewer bone metastatic lesions compared to the shSCM PC3ML cells (Fig. 3d and f).

### Axl inhibition impairs osteoclast formation

**Fig. 4:**
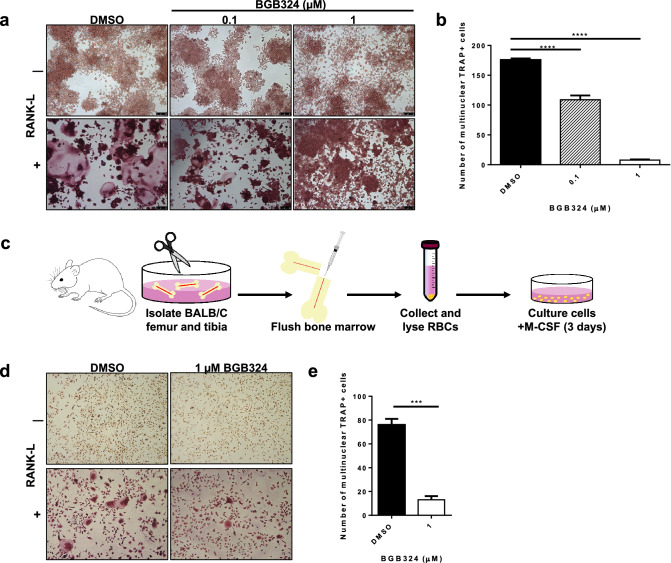
Pharmacologic inhibition of Axl inhibits osteoclast formation. Osteoclast progenitor cells were seeded in a 24-well plate (2 × 10^4^ cells/well), treated with or without 35 ng/ml RANK-L and varying concentrations of BGB324. Five days later, mature and differentiated osteoclasts were fixed and stained for tartrate resistant acid phosphatase (TRAP). **a** Representative images of TRAP-positive, multinucleated osteoclasts, 5X magnification. **b** Total number of TRAP-positive, multinucleated ($\ge$3 nuclei) osteoclasts, analyzed by One-way ANOVA. **c** Scheme of isolating primary osteoclast precursor cells from 6–8 week-old female BALB/c mice. **d** Representative images of TRAP-positive, multinucleated osteoclasts differentiated from primary osteoclast precursor cells of BALB/c mice, 5X magnification. **e** Total number of TRAP-positive, multinucleated ($\ge$3 nuclei) osteoclasts differentiated from primary osteoclast precursor cells, analyzed by Student’s *t-*test. Results are the mean and standard error values of three independent experiments. ***p < 0.0001, ****p < 0.00001

As metastatic bone lesions are often characterized by excess number and activity of osteoclasts, we evaluated the role of Axl in osteoclasts by stimulating the preosteoclast cells with receptor activator of nuclear factor κ-B ligand (RANK-L) to differentiate preosteoclasts to mature osteoclasts. Treatment of murine preosteoclast cell line (Raw264.7) with BGB324 formed significantly less mature, multinucleated osteoclasts compared to the preosteoclast cells treated with the DMSO vehicle control, upon treatment with RANK-L (Fig. 4a and b). In addition, the efficacy of BGB324 on osteoclastogenesis was evaluated on bone marrow derived cells (BMDCs) isolated from female BALB/c mice (Fig. 4c). In the presence of BGB324, the BMDCs showed impaired ability to differentiate into mature, multinucleated osteoclasts upon RANK-L treatment compared to its vehicle treated control BMDCs (Fig. 4d and e).

**Fig. 5:**
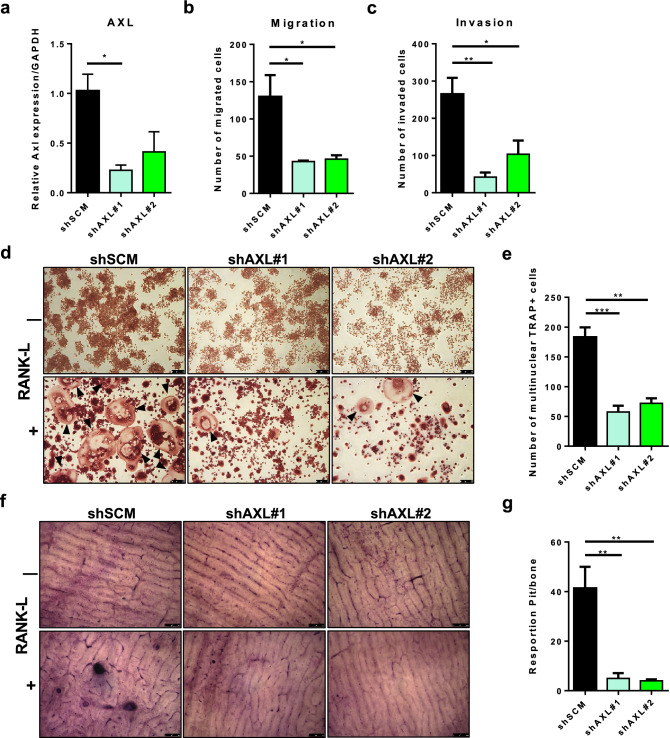
Axl-expressing osteoclast precursor cells promote osteoclastogenesis. **a** Axl was genetically inactivated by shRNA in murine osteoclast progenitor cell line (Raw264.7), and two knockdown clones (shAXL#1 and shAXL#2) were selected by qPCR for further studies. shSCM and shAXL osteoclast progenitor cells were seeded in the transwell migration or invasion chambers. After 24 h, the number of migrated **b** Or invaded **c** Cells were counted. shSCM or shAXL osteoclast progenitor cells (2 × 10^4^ cells/well of a 24-well plate) were treated with 35 ng/ml RANK-L. Five days later, mature and differentiated osteoclasts were fixed and stained for tartrate resistant acid phosphatase (TRAP). **d** Representative images of TRAP-positive, multinucleated osteoclasts (indicated by the black arrows), 5X magnification. **e** Total number of TRAP-positive, multinucleated ($\ge$3 nuclei) osteoclasts were quantified. shSCM or shAXL osteoclast progenitor cells (2 × 10^4^ cells/well of a 24-well plate) were seeded on a dentine slice treated with or without 35 ng/ml RANK-L. Five days later, dentine slices were stained with toluidine blue solution to observe resorption pits on a dentine slice. **f** Representative images of bone slices from shSCM and shAXL cells treated with or without RANK-L, 10X magnification. **g** Total number of resorbed pits per bone slice were quantified.(*p < 0.05, **p < 0.01, ***p < 0.0001

In addition, Raw264.7 cells were transduced with lentiviral shRNA against scrambled non-target sequence (shSCM) or AXL (shAXL), and the knockdown efficiency was confirmed by qPCR (Fig. 5a). Axl knockdown preosteoclast cells showed significantly impaired motility and invasiveness in transwell chamber assays (Fig. 5b and c). Axl knockdown preosteoclast cells formed significantly less mature, multinucleated osteoclasts compared to shSCM preosteoclast cells upon treatment with RANK-L (Fig. 5d and e). Furthermore, the shAXL preosteoclasts showed significantly impaired capacity to resorb bones slices compared to shSCM preosteoclasts upon treatment with RANK-L (Fig. 5f and g).

### Axl knockdown decreases MCP-1 secretion

**Fig. 6:**
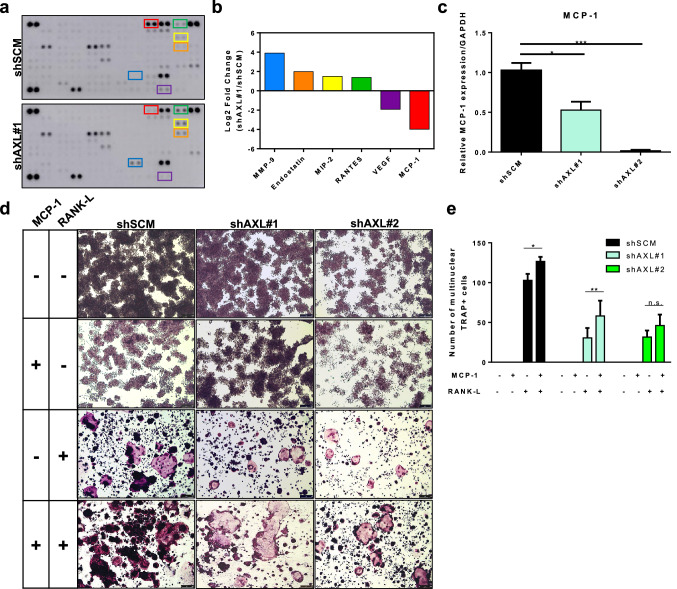
MCP-1 is a downstream effector of Axl in osteoclast precursor cells. Conditioned media of shSCM and shAXL osteoclast progenitor cells were collected and analyzed by cytokine array (**a**, **b**). **c** MCP-1 expression in shSCM and shAXL osteoclast progenitor cells were assessed by qPCR. Osteoclast progenitor cells were seeded and treated with or without 35 ng/ml RANK-L and/or 50 ng/ml MCP-1. Five days later, mature and differentiated osteoclasts were fixed and stained for tartrate resistant acid phosphatase (TRAP). **d** Representative images of TRAP-positive, multinucleated osteoclasts, 5X magnification. **e** Total number of TRAP-positive, multinucleated ($\ge$3 nuclei) osteoclasts were quantified. *p < 0.05, **p < 0.01, ***p < 0.0001

To determine the downstream effectors of the Axl pathway in preosteoclast cells, conditioned media collected from shSCM or shAXL preosteoclast cells were analyzed using a cytokine array. Comparison of shSCM and shAXL conditioned media revealed six proteins whose secreted levels was changed more than twofold in shAXL compared with shSCM (Fig. 6a and b). Monocyte chemoattractant protein 1 (MCP-1) secretion was 15.7 times lower in shAXL conditioned medium than in shSCM conditioned medium (Fig. 6b). The decrease in MCP-1 secretion was associated with a significantly lower expression of MCP-1 in shAXL preosteoclast cells compared to shSCM preosteoclast cells (Fig. 6c).

### Exogenous MCP-1 increases osteoclast formation

As MCP-1 has been previously implicated to promote osteoclast formation [38], we evaluated whether exogenous MCP-1 would rescue RANK-L-induced osteoclast formation in shAXL preosteoclasts. MCP-1 alone did not induce osteoclast formation in preosteoclasts, but combination of MCP-1 and RANK-L increased the number of osteoclasts in both shSCM and shAXL cells (Fig. 6d and e).

## Discussion

Previous studies have shown that Axl promotes multiple aspects of the metastatic phenotypes, including migration, invasion, and survival, in colorectal, renal, and osteosarcoma cells [39–43]. Our present study confirms the expression of Axl in human triple negative breast (MDA-MB-231) and prostate (PC3ML) cancer cells. Axl knockdown in MDA-MB-231 and PC3ML cells significantly impaired metastatic phenotypes, including migration and invasion (Fig. 1).

Given that bone is the most common site of metastasis for breast and prostate cancers, we evaluated the effects of Axl knockdown tumor cells on bone colonization *in vivo*. Our findings indicate that Axl inhibition impairs breast and prostate cancer cell bone metastasis (Fig. 3). A critical factor in bone metastasis is tumor cell-induced bone remodeling, also known as the vicious cycle. Relocation of tumor cells to bones disrupts the normal homeostatic regulations of bone formation and degradation by osteoblasts and osteoclasts, respectively [12, 44]. Metastatic bone lesions are characterized abundant and hyperactive osteoclasts, which demineralize and degrade the bones to promote the growth of tumor cells in the resorbed cavity [6, 45]. These osteoclasts arise from an osteoclast precursor cell from a monocytic lineage, which also expresses Axl [31, 32]. Our findings suggest that Axl expressed by osteoclast precursors contribute to osteoclast maturation and differentiation upon stimulation with RANK-L (Fig. 3). Although the underlying mechanism for a role of Axl in osteoclast maturation remains unknown, recent studies have demonstrated that Tyro3 and fibroblast growth factor receptor type 1 (FGFR1) in mature osteoclasts stimulate osteoclast-mediated bone resorption [46, 47], support this notion.

We demonstrated that the conditioned medium of shAXL preosteoclast cells revealed six differentially secreted proteins (four upregulated and two downregulated). In support of our previous report on the role of the Axl signaling on angiogenesis [33], we observed an increased secretion of endostatin, an endogenous inhibitor of angiogenesis [48], and decreased secretion of VEGF (Fig. 6a, b). Another potential downstream effector of the Axl pathway is MCP-1. The shAXL cells expressed and secreted significantly lower levels of MCP-1 than shSCM cells (Fig. 6b, c). MCP-1 expression has been associated with several pathological conditions, including rheumatoid arthritis, multiple sclerosis, and tumor-induced bone loss [49]. Furthermore, Miyamoto and colleagues demonstrated that osteoclast progenitor cells isolated from MCP-1-deficient mice formed significantly less mature osteoclasts compared to osteoclast progenitor cells isolated from wild-type mice, upon treatment with RANK-L [38]. Our findings suggest that MCP-1 may be a downstream effector of Axl pathway in the osteoclast progenitor cells. While MMP-9 has been associated with the metastatic cascade in breast cancer setting [50, 51] and the Axl signaling pathway [52], our study demonstrated that MMP-9 secretion increased in the shAXL Raw264.7 cells. Further studies are warranted to understand the downstream molecular signaling pathway and effectors of Axl. Nonetheless, therapeutic targeting of the Axl pathway may have utility to impair tumor cell functions and tumor-induced bone remodeling.

Receptor tyrosine kinases are frequently mutated, amplified, or overexpressed in different cancer types and are known drive many steps of the metastatic cascade, including migration, invasion, proliferation, survival and angiogenesis. Altered expressions of receptor tyrosine kinases also are associated with therapeutic resistance, poor clinical outcome, and metastatic disease. Hence, a number of specific and pan-receptor tyrosine kinase inhibitors have been developed to impair cancer progression and metastasis. Bemcentinib (BGB324), a specific type I kinase inhibitor of Axl with promising preclinical results [53] has entered clinical trials for cancers including non-small cell lung carcinoma, adenocarcinoma of the lung, acute myeloid leukemia, melanoma and pancreatic cancer (obtained from ClinicalTrials.gov). Our studies showed that pharmacologic Axl inhibition by BGB324 impaired tumor cell migration and invasion (Fig. 2). In addition to its effects on tumor cells, we demonstrated that BGB324 impaired RANK-L-induced osteoclast maturation and differentiation in osteoclast precursor cells and BMDCs derived from female BALB/c mice (Fig. 4). Taken together, we predict that pharmacologic inhibition of Axl will target neoplastic as well as stromal cells in the tumor microenvironment and hence impair tumor progression and metastasis.

Growth arrest specific 6 (Gas6) protein is one of the ligands for Axl. A recent study by Ibrahim et al*.* demonstrated that elevated *GAS6* expression does not predict for breast cancer outcomes [54]. However, Gas6 has been shown to increase formation of bone resorption pits and promote proliferation of tumor cells [46, 55]. In the bone microenvironment, Shiozawa and colleagues demonstrated that Gas6 may be more abundantly secreted in human osteoblasts than osteosarcoma cells [55]. It is therefore possible that Gas6 is involved in the homing of tumor cells to the bone microenvironment. Once tumor cells colonize the bone, tumor cells may further induce Gas6 expression and secretion by bone marrow derived macrophages (BMDMs) to further promote tumor growth and bone remodeling [46, 56, 57]. Although the present study did not examine the role of Gas6 on prostate and breast cancer cells, we predict that Gas6 is an important ligand that mediates autocrine and paracrine signaling axes between neoplastic and stromal cells. Future studies include characterization of the tumor immune microenvironment in the primary and disseminated tumors to understand multimodal roles of Axl in both neoplastic and host stromal cells.

Overall, the present results demonstrate that the Axl signaling pathway in both neoplastic and host cells may significantly impact multiple vital steps associated with the successful establishment of secondary tumor foci. Inhibition of Axl impaired tumor cell migration, invasion, and tumor cell-induced angiogenesis. In osteoclast progenitor cells, genetic and pharmacologic inhibition of Axl inhibited osteoclast maturation and differentiation. In addition, a previous study has shown that 20G7-D9, a specific anti-human AXL murine IgG1 monoclonal antibody, impaired tumor growth and metastatic spread of triple negative breast cancer cell lines [58, 59]. Collectively, the present data suggest that Axl is a promising therapeutic target that may impair bone metastasis by interfering with the Axl signaling axis in both neoplastic and host cells thus affecting neoplastic cell processes including migration, invasion, as well as osteoclastogenesis.

## Supplementary Information

Below is the link to the electronic supplementary material.

Supplementary file1 (PDF 162 kb)

## Data Availability

The raw data obtained and analyzed from this study are available from the corresponding author upon request.

## Abbreviations

BLI: Bioluminescent imaging
GAPDH: Glyceraldehyde 3-phosphate dehydrogenase
Gas6: Growth arrest specific 6
MCP-1: Monocyte chemoattractant protein 1
RANK-L: Receptor activator of nuclear factor κ-B ligand
RTK: Receptor tyrosine kinase
shRNA: Short hairpin RNA
TRAP: Tartrate resistant acid phosphatase
