# Supplementary material for: Inhibition of the Axl pathway impairs breast and prostate cancer metastasis to the bones and bone remodeling
Source: Clin Exp Metastasis. 2021 Mar 31;38(3):321–35. doi: 10.1007/s10585-021-10093-z (PMC8179919; doi:10.1007/s10585-021-10093-z)

Supplementary Fig 1. Axl KD does not affect tumor cell growth or proliferation.

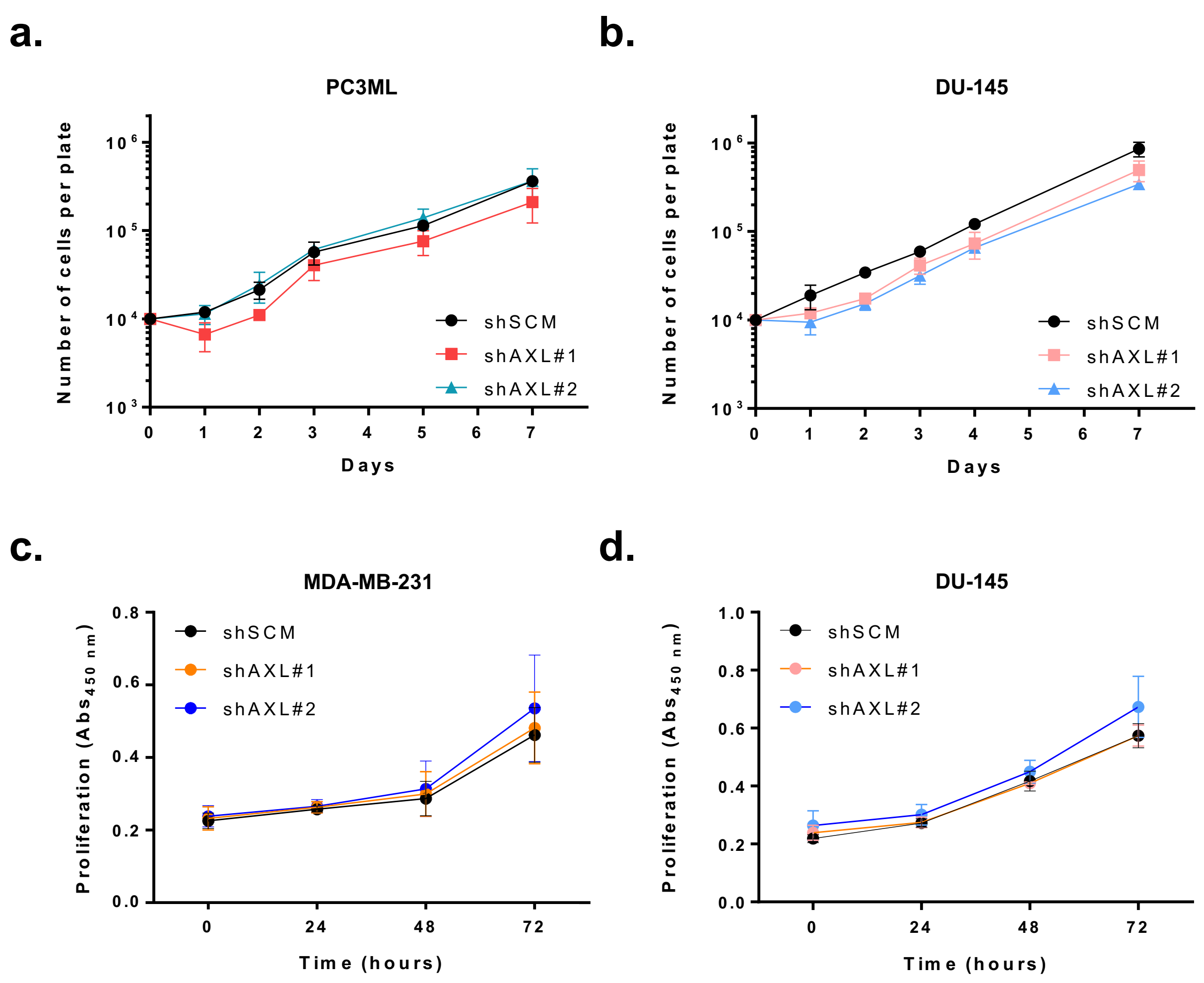

Supplementary Fig. 2 Pharmacologic Axl inhibitor BGB324 does not impair tumor cell proliferation.

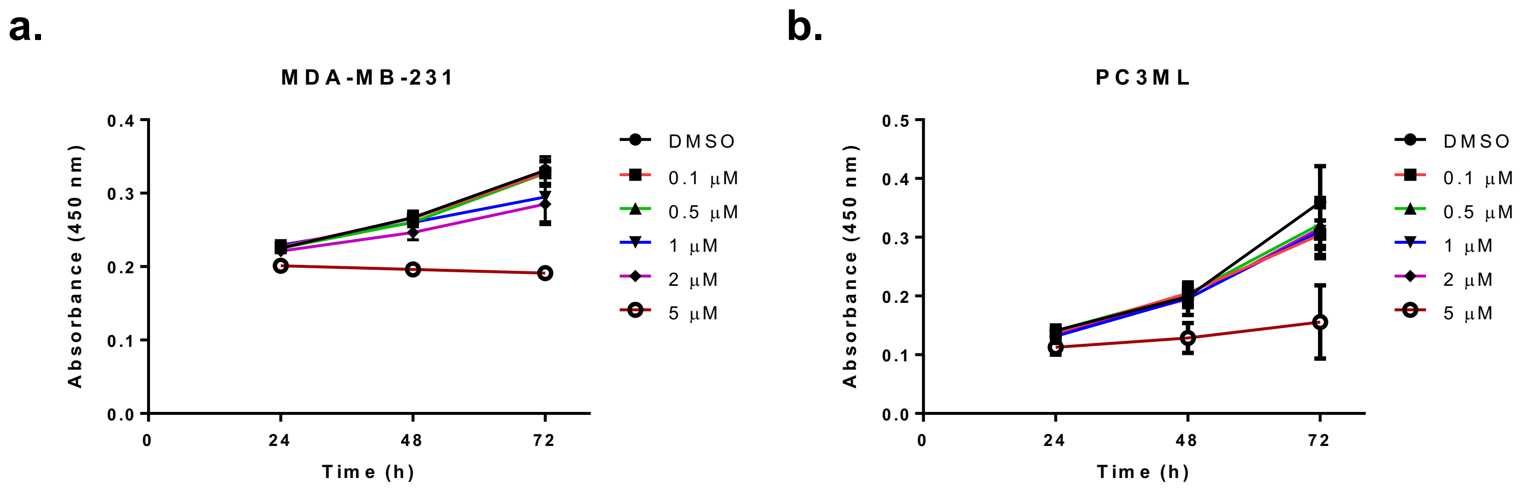

Supplement: Supplementary file 1 — Supplementary file1 (PDF 162 kb) [file 10585_2021_10093_MOESM1_ESM.pdf]
